# Pennsylvania Hospital—Surgical Wards—Service of Dr. Norris

**Published:** 1842-03-12

**Authors:** E. Hartshorne

**Affiliations:** Resident


### CLINICAL REPORTS.

*Pennsylvania Hospital—Surgical Wards—Service of Dr. Norris.*
By Dr. E. Hartshorne, Resident.

1. Micajah D., aged 51, of a rather muscular frame, entered Jan. 19, for a luxation of the right humerus into the axilla, of twenty-eight days duration. The bone was displaced by a blow upon the shoulder in a fall of some feet. Violent inflammation of the joint soon followed the reception of the injury, and did not entirely subside for several days. Meanwhile reduction of the limb was not attempted until the twenty-third day, when an unsuccessful trial was made by a country practitioner in the neighbourhood. When presented at the hospital, the shoulder, then free from inflammation, exhibited, in a striking degree, every characteristic of this kind of dislocation. The sharp angle caused by the projecting acromion, the depression, immediately below the latter, the emaciated and nearly powerless deltoid and other muscles of the shoulder, the head of the bone in the axilla, the impeded motions of the arm, and especially the impossibility of adducting the elbow to the body, were all distinctly observed. The luxation was reduced the day of his admission, under the direction of Dr. Norris, in thirty-five minutes; during which time the man took a grain and a half of tartarised antimony, having previously taken two doses of half a grain each in the course of the half hour immediately preceding the operation. The patient having been seated on a low stool, his body was fixed by means of a folded sheet, passed horizontally around his chest, below the axilla of the affected limb, and fastened to a staple a short distance to the left.

Extension was affected by means of the pulling tackles attached on a level with the shoulder to a staple on the right, and connected with a roller towel, which was securely bound with a wet bandage to the right arm above the elbow. Counter-extension of the acromion was sustained by an assistant with a roller towel passing round his body, and just beneath the margin of the process, on which he drew by leaning back upon the towel. The head of the bone was lifted upwards, in the latter stage of the operation, by another assistant, drawing with a towel from above.

After regular and continued extension for thirty minutes, during which the arm was at intervals carefully moved and rotated by the directing operator, the rope was dropped, and the outstretched extremity suddenly brought down to the side of the body. This manœuvre partially restored the bone, which was again slightly displaced, but was immediately reduced by traction without the rope and pulleys. The arm was then suspended in the sling of Fox's clavicle apparatus. The whole operation occupied thirty-five minutes, the latter five minutes having been consumed in adjusting the bone after the continued extension had ceased. Fox's apparatus was worn two days, during which the injured part was occasionally bathed in warm soap liniment and laudanum. After the second day a handkerchief sling alone was used, and the joint was passively exercised every day. The patient was discharged on the sixth day, Jan. 25, at his own request, the joint being perfectly good, although, of course, still somewhat sore and rigid.

